# Potential for England’s statutory school entry assessment to identify special educational needs and reveal structural inequalities: a population-based study

**DOI:** 10.1136/archdischild-2023-325590

**Published:** 2023-10-12

**Authors:** Megan L Wood, Lydia Gunning, Sam Relins, Kuldeep Sohal, John Wright, Mark Mon-Williams, Amy L Atkinson

**Affiliations:** 1 School of Psychology, University of Leeds, Leeds, UK; 2 Bradford Institute for Health Research, Bradford, UK; 3 Department of Psychology, Lancaster University, Lancaster, UK

**Keywords:** Child Development, Child Health, Psychology, Mental health

## Abstract

**Objective:**

To investigate at a population level whether England’s universal assessment of ‘school readiness’ is associated with later identification of special educational needs (SEN). Also, whether ethnic differences exist in SEN identification (white British versus ethnic minority) and whether this varies as a function of school readiness.

**Method:**

Analysis included 53 229 individuals aged 5–12 years from the Connected Bradford Database (2012/2013–2019/2020). Logistic regression analyses examined: (1) whether reaching a ‘good level of development’ on England’s ‘school readiness’ assessment was associated with later SEN identification; and (2) whether interactions exist between school readiness and ethnicity.

**Results:**

32 515 of 53 229 children reached a good level of development, of which 3036 (9.3%) were identified as having SEN. In contrast, 10 171 of 20 714 (49.1%) of children who did not reach a good level of development were later identified as having SEN. Children not reaching a good level of development had increased odds of being later identified as having SEN after controlling for covariates (OR: 8.50, 95% CI: 8.10 to 8.91). In children who did not reach a good level of development, white British children had higher odds of being identified as having SEN compared with ethnic minority peers (OR: 1.22, 95% CI: 1.11 to 1.34). No ethnic differences of having SEN were observed in children reaching a good level of development (OR: 1.04, 95% CI: 0.93 to 1.16).

**Conclusions:**

School readiness assessments are associated with later SEN identification. Structural inequalities may exist in SEN identification in children not entering formal education ‘school ready’. Such assessments could facilitate earlier identification of SEN and reduce structural inequalities in its identification.

**WHAT IS ALREADY KNOWN ON THIS TOPIC**

- England’s school entry assessments can identify children at risk of special educational needs (SEN).
- These assessments can also identify children at risk of poor academic attainment.

**WHAT THIS STUDY ADDS**

- The potential of using statutory school entry assessments to identify children at increased risk of being identified as having SEN at a population-level.
- Structural inequalities may exist in the identification of SEN in children who do not enter formal education ‘school ready’

**HOW THIS STUDY MIGHT AFFECT RESEARCH, PRACTICE OR POLICY**

- Using school entry assessments as a screening tool may facilitate earlier identification of SEN.
- Address structural inequalities by identifying children who could benefit for further monitoring or assessment who may otherwise ‘fall through the net’.

## Introduction

Identification of special educational needs (SEN) is a vital first step to ensuring children get the necessary support to enable them to thrive in school. In the UK, SEN is defined as ‘a learning difficulty or disability which calls for special educational provision to be made’^1^ which includes, but not limited to, ‘autism; social, emotional and mental health needs; or moderate learning difficulties’. Many children with SEN are not identified for some years into their formal education,^2^ despite presenting with indicators as early as infancy.^3^ These delays can result in a range of negative outcomes, including disengagement,^4 5^ school exclusion^6^ and poor academic achievement^5^ with consequent long-term physical and mental health problems.^7^ Thus, there is a need to identify children with SEN early.^8 9^

School readiness evaluations are frequently conducted at school entry in various countries (eg, England, Australia, Canada^8 10 11^). As part of these assessments, children are typically evaluated on a range of academic (eg, numeracy) and non-academic abilities (eg, social skills^12 13^). As children with SEN often experience difficulties in these areas^8^ (see O’Connor *et al* 5 for a conceptual model), school readiness evaluations may identify children at increased risk of needing support.^8^ Indeed, there is accumulating evidence that school readiness evaluations can identify children at increased risk of various developmental difficulties, including autism^14^ and dyslexia.^15^ More broadly, previous research has also revealed associations between school readiness and SEN at school entry.^13 16^

Atkinson *et al* 8 used data from the Born in Bradford longitudinal cohort to examine whether the school readiness assessment conducted universally in England (the Early Years Foundation Stage Profile; EYFSP) could identify children at risk of later needing support. A strong relationship was observed, suggesting that school entry assessments could be used as a tool to identify children who are at increased risk of needing SEN support but currently ‘under the radar’ (see also Hughes *et al* 9).

A range of demographic factors have also been found to be associated with identification of SEN, which may reflect both risk factors in requiring additional need and structural inequalities in the needs being identified. For example, previous research suggests that children from more disadvantaged backgrounds are at increased risk of requiring additional support.^1 17 18^ This may be influenced by features more common in communities with lower socioeconomic position such as increased parental ill-health and drug/alcohol dependency.^19^ Conversely, research has shown that children from ethnic minority backgrounds are less likely to have SEN, but this may reflect structural inequalities in identification.^20–22^ These structural inequalities may arise due to variation in views relating to child development between cultures^23^ or knowledge of and access to services and support within ethnic minority families.^20^ To date, no existing research has investigated whether such ethnic differences vary as a function of school readiness. This would indicate whether structural inequalities relating to ethnicity are present universally, or whether their effects are limited to particular groups (eg, children who enter formal education not being ‘school ready’).

The current study had two aims. First, it aimed to use population-level data to test Atkinson *et al*’s^8^ suggestion that school readiness evaluations could be used to identify SEN, after controlling for covariates (eg, sex, ethnicity).^24^ Second, it aimed to investigate whether ethnic differences exist in the identification of SEN, and whether this differs as a function of school readiness. We used data from the Connected Bradford dataset^24^ to investigate this.

## Method

### Study design, setting and participants

The study design was longitudinal. Data were collated from the Connected Bradford population-level linked database for over 800 000 citizens across the Bradford district, UK.^24^ Education records provided by the Department for Education were linked across multiple time points, spanning academic years 2012–2013 to 2019–2020. Data were extracted in April 2021. Children included in the sample were aged 5 (year 1) to 12 (year 7) years old. See online supplemental materials 1 for a description of how the sample was derived. Sample demographics of the final sample in relation to the wider Connected Bradford database are included in online supplemental materials 2.

10.1136/archdischild-2023-325590.supp1 Supplementary data

### Variables

#### Outcome variable

SEN status (yes/no) was obtained from the Department for Education records and reflected whether children had ever been identified as having SEN. Children were identified as having SEN if they had ever been recorded as having a School Action plan, a School Action Plus plan, an SEN statement, an Education, Health and Care plan, or in receipt of SEN support.

#### Predictor variables

We used data from the school readiness assessment conducted universally in England (the EYFSP) in the first year of school (‘reception’; 4–5 years of age). The assessment allowed us to differentiate between children who were judged by teachers as being ‘school ready’, having reached a ‘good level of development’ (an outcome measure within the EYFSP) and those who were not (having not reached a ‘good level of development’).^8^ We focused our analyses on the post-2013 England school readiness assessments as a different version was used before this (see online supplemental materials 3 for further detail).

For ethnicity, children were categorised as either ‘white British’ or ‘ethnic minority’ based on family-reported census records (see online supplemental materials 4 for a further breakdown). Sex (female, male) was also taken from family-reported census records. Eligibility for free school meals (no/yes) was used as a proxy for socioeconomic position as children from lower socioeconomic position households are eligible for free school meals in the UK. This was obtained from Department for Education records. English as an additional language status (no/yes) was teacher-reported and obtained from the Department for Education records.

### Study size

To be included in analyses, individuals were required to have complete data for all variables. Further, as our focus was on children who fall ‘under the radar’, we removed children already identified as having SEN during reception (ie, before or at the same time as the school readiness assessment; determined by Department for Education records).

### Statistical methods

We conducted logistic regression analysis to investigate whether the good level of development (reached, not reached) was associated with later SEN identification (no, yes). An unadjusted logistic regression model was first conducted, followed by a model adjusting for ethnicity (ethnic minority, white British), eligibility for free school meals (no, yes), sex (female, male) and English as an additional language status (no, yes). Sensitivity, specificity, positive predictive value, negative predictive value and classification rates were also calculated to assess the potential of school entry assessments for flagging unidentified SEN.

We then investigated whether interactions exist between the good level of development and ethnicity. A logistic regression model was conducted with good level of development, ethnicity and their interaction, as predictors. This analysis controlled for eligibility for free school meals, sex and English as an additional language status. The reference category for good level of development was ‘reached’, while the reference category for ethnicity was ‘ethnic minority’. Significant interactions were followed up with planned comparisons (corrected using Bonferroni) to investigate whether an effect of ethnicity was present in children who did, and who did not, reach a good level of development.

Analyses were conducted in R (V.4.0.2; see online supplemental materials 5 for R code).^25^ Participants with missing data for any variable were excluded. In line with previous research (eg, 8 26), we report ORs.

## Results

The final sample contained data for 53 229 children (see online supplemental materials 1 and 2 for derivation). Table 1 displays the sample characteristics.

**Table 1:** Sample demographics

	Whole sample (n=53 229)	SEN (n=13 207)	Not SEN (n=40 022)
SEN			
Yes	13 207 (24.8%)	–	–
No	40 022 (75.2%)	–	–
Good level of development			
Reached	32 515 (61.1%)	3036 (23.0%)	29 479 (73.7%)
Not reached	20 714 (38.9%)	10 171 (77.0%)	10 543 (26.3%)
Ethnicity			
White British	27 458 (51.6%)	6822 (51.7%)	20 636 (51.6%)
Minority	25 771 (48.4%)	6385 (48.3%)	19 386 (48.4%)
Free school meal eligibility*			
Yes	13 658 (25.7%)	4983 (37.7%)	8675 (21.7%)
No	39 571 (74.3%)	8224 (62.3%)	31 347 (78.3%)
Sex			
Male	26 583 (49.9%)	8382 (63.5%)	18 201 (45.5%)
Female	26 646 (50.1%)	4825 (36.5%)	21 821 (54.5%)
English as an additional language			
Yes	18 914 (35.5%)	4876 (36.9%)	14 038 (35.1%)
No	34 315 (64.5%)	8331 (63.1%)	25 984 (64.9%)

Percentages may not add up to 100% due to rounding.

*Proxy for socioeconomic position.

SEN, special educational needs.

### Is school readiness associated with SEN status?

The frequencies and percentages of children identified as having SEN, depending on whether they reached a good level of development, are displayed in table 2.

**Table 2:** The number of children identified as having special educational needs (SEN) after reception (yes/no) as a function of the good level of development (GLD) measure (reached/not reached)

GLD	Identified as SEN*		Total
	Yes	No	
Reached	3036 (9.3%)	29 479 (90.7%)	32 515 (100%)
Not reached	10 171 (49.1%)	10 543 (50.9%)	20 714 (100%)
Total	13 207	40 022	53 229

*After reception year.

**Figure 1:**
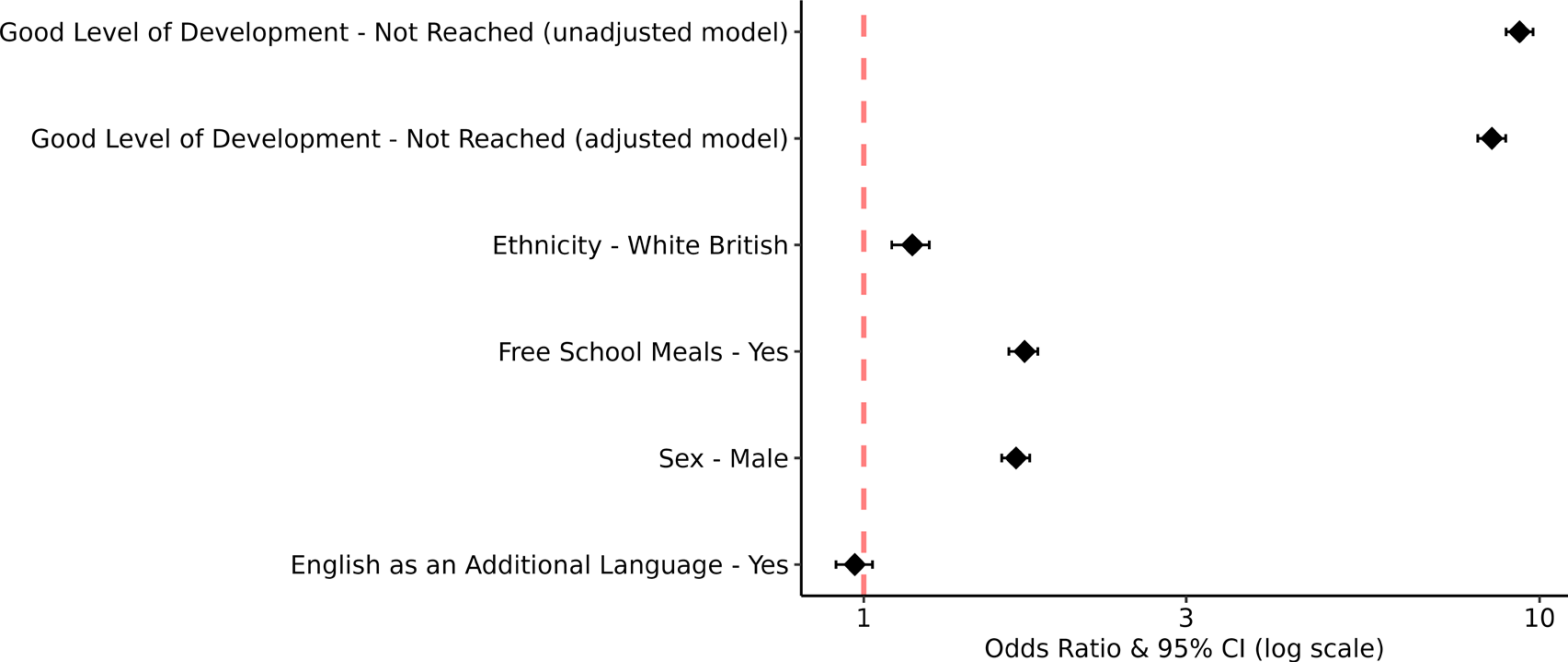
OR and 95% CIs for the association between GLD and SEN status (unadjusted model) and GLD+ethnicity+free school meal eligibility+sex+English as an additional language status (adjusted model). GLD, good level of development; SEN, special educational needs.

The outcomes from the logistic regression models examining whether reaching a good level of development is associated with SEN are presented in figure 1. Children who did not reach a good level of development had higher odds of being identified as having SEN compared with children who reached a good level of development. This was observed in both the unadjusted (OR: 9.34, 95% CI: 8.92 to 9.78, p<0.001) and adjusted (OR: 8.50, 95% CI: 8.10 to 8.91, p<0.001) models. Several covariates were also associated with SEN status. Children in receipt of free school meals had higher odds of being identified as having SEN compared with children not in receipt of free school meals (OR: 1.73, 95% CI: 1.64 to 1.81, p<0.001), and males had increased odds of being identified as having SEN compared with females (OR: 1.68, 95% CI: 1.60 to 1.76, p<0.001). Furthermore, white British children had increased odds of being identified as having SEN relative to children from ethnic minorities (OR: 1.18, 95% CI: 1.10 to 1.25, p<0.001).

#### Classification rates

Classification rates are displayed in table 3. Positive predictive value was relatively low, with approximately one in two children who did not reach a good level of development later identified as having SEN. However, negative predictive value was high, indicating few children who reached a good level of development were identified as having SEN.

**Table 3:** Classification rates for SEN as the outcome variable

Classification rate	Definition	Value	95% CIs
Sensitivity	% of children identified as having SEN without good development	0.77	0.76 to 0.78
Specificity	% of children not identified as having SEN with good development	0.74	0.73 to 0.74
Positive predictive value	Probability a child who did not reach good development being identified as having SEN	0.49	0.48 to 0.50
Negative predictive value	Probability a child who reached good development was not identified as having SEN	0.91	0.90 to 0.91
Correct classification rates	% of children correctly classified as at risk/not at risk of being identified as having SEN based on school readiness	0.74	0.74 to 0.75

SEN, special educational needs.

### Do school readiness and ethnicity interact regarding SEN identification?

**Figure 2:**
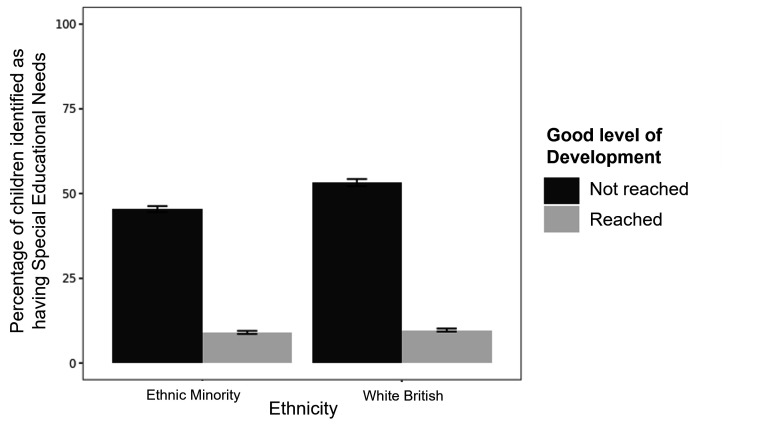
Percentage of children identified as having special educational needs by good level of development and ethnicity. Error bars show 95% CIs.

The percentages of children identified as having SEN are displayed in figure 2, as a function of good level of development and ethnicity.

The logistic regression model revealed a significant interaction between reaching a good level of development and ethnicity after controlling for covariates (OR: 1.18, 95% CI: 1.07 to 1.29, p<0.001; see online supplemental materials 6 for full reporting). Post-hoc tests revealed no ethnic differences in children who reached a good level of development (OR: 1.04, 95% CI: 0.93 to 1.16, p=1.00). In children who did not reach a good level of development, white British children had increased odds of being identified as SEN compared with children from ethnic minority backgrounds (OR: 1.22, 95% CI: 1.11 to 1.34, p<0.001).

## Discussion

In a population sample, we found that children assessed as not being ‘school ready’ had higher odds of being identified as having SEN compared with children who were ‘school ready’. We then examined whether the extent to which ethnicity was associated with SEN differed as a function of school readiness. This analysis revealed no ethnic differences in children who reached a good level of development. In contrast, for children who did not reach a good level of development, white British children had higher odds of being identified as having SEN compared with children from ethnic minority backgrounds.

Evidence that reaching a good level of development was associated with later SEN status illustrates that England’s school readiness assessments can identify children who are at increased risk of being identified as having SEN.^8 14^ Such findings have important implications for policymakers, schools, children and their families. First, identifying children ‘at risk’ earlier means targeted interventions can be implemented within schools to provide children with the support they need to thrive. Second, the use of an early ‘screener’ allows at-risk children to be identified before they exhibit symptoms of a diagnosable learning difficulty. Thus, school readiness assessments can allow support to be put into place before more serious difficulties occur and prevent a child falling behind their peers.^27^ It is, however, worth noting that the assessment does appear to overidentify the number of children at risk, with positive predictive value for the assessment being relatively low (0.49; see also 8 9). We therefore suggest that outcomes from school entry assessments should be considered alongside other information, such as the current teacher’s judgement.^8^

White British children who were not ‘school ready’ had higher odds of being identified as having SEN relative to ethnic minority peers who were not ‘school ready’. This is in line with previous research which has found ethnic biases in smaller samples.^20–22^ More broadly, this adds to a growing body of literature indicating that individuals from ethnic minority backgrounds are identified later than their ethnic majority counterparts in conditions as diverse as autism,^28^ tuberculosis^29^ and cancer.^30^ Importantly, however, there were no ethnic differences in children who entered formal education ‘school ready’, thus indicating that the association between ethnicity and SEN is not universal.

Why might ethnic differences have only emerged in children who were not ‘school ready’? One possibility is that families of children from ethnic minority backgrounds who do not enter formal education ‘school ready’ may be more likely to lack understanding of how to navigate complex systems (eg, schools, the SEN support system) relative to other groups.^31^ Another possibility is that difficulties at school entry in children from ethnic minority backgrounds could be attributed to factors other than SEN (eg, cultural factors). Use of school readiness assessments to flag these children as ‘at risk’ would facilitate further monitoring or assessments. This may reduce structural inequalities in the identification of SEN. It would be beneficial for further research to investigate this possibility.

There are several limitations with this study. First, the findings are specific to a single district in England (Bradford), which has a large ethnic diversity compared with the rest of the country.^32^ Future work should therefore examine this at a national level. Furthermore, the current analyses determined the odds of a child later being *identified* as having SEN. It does not consider the children who were never formally identified. More research is required to determine whether there are structural inequalities in the children who remain ‘under the radar’. In addition, it is possible that other confounding variables exist which were not controlled for. Finally, while this research and previous studies (eg,^8 14^) have shown the utility of England’s school readiness assessment, it is possible that it may not be *as* effective at identifying difficulties in all groups of children. For example, it may be difficult to judge domains such as social skills and literacy in children with lower English proficiency^33^ (although English as an additional language status was controlled for in the present analyses). Teacher-reported assessments may also be subject to subtle ethnic stereotypes.^11 34^ Future research should examine these possibilities in relation to school readiness evaluations. Additional work may also consider the longer-term associations between school readiness and other aspects of learning and development, such as career prospects and Not in Education, Employment or Training status.

In conclusion, using population-level data, this study indicates that a potentially powerful tool already exists that can address the pressing international need to improve early identification of SEN. It also suggests ethnic differences exist in the identification of SEN in children who do not enter formal education ‘school ready’. Using school readiness assessments may help to address this, by identifying subgroups of children at school entry who may particularly benefit from additional monitoring.

## Data Availability

Data are available upon reasonable request.
