# Supplementary material for: Potential for England’s statutory school entry assessment to identify special educational needs and reveal structural inequalities: a population-based study
Source: Arch Dis Child. 2023 Oct 12;109(1):52–7. doi: 10.1136/archdischild-2023-325590 (PMC10804042; doi:10.1136/archdischild-2023-325590)
Supplement: Supplementary data [file archdischild-2023-325590supp001.pdf]

Supplementary Materials

1. **Flowchart of how the sample was derived. Note that arrows pointing towards the right**

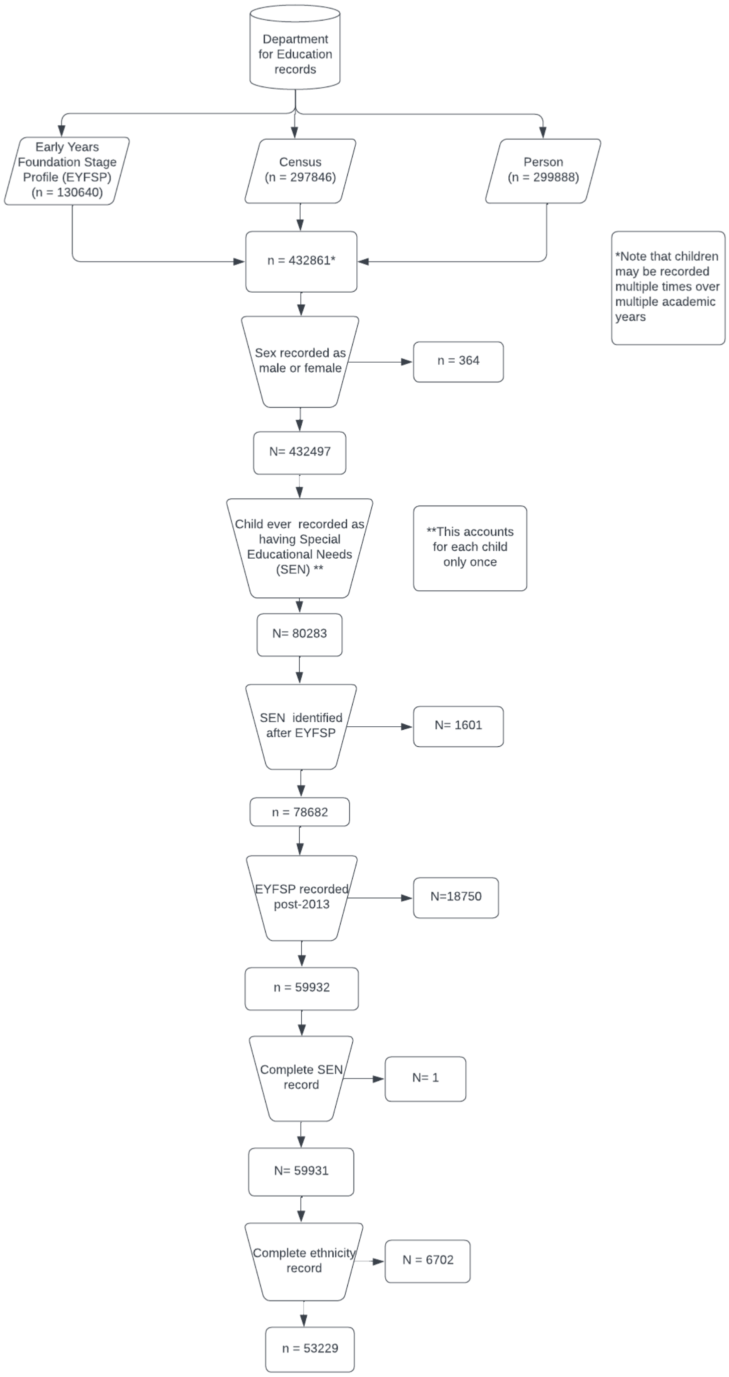

indicated excluded participants.

2. Sample demographics compared to wider population in Connected Bradford

	Included Sample (n=53266)	Whole Connected Bradford Cohort (n=130552)
SEN		
Yes	13207 (24.8%)	42777 (32.8%)
No	40022 (75.2%)	87456 (67.0%)
Missing	-	319 (0.2%)
GLD		
Yes	32515 (61.1%)	49260 (37.7%)
No	20714 (38.9%)	31571 (24.2%)
Missing	-	49721 (38.1%)
Ethnicity		
White British	27458 (51.6%)	57647 (44.2%)
Minority	25771 (48.4%)	52762 (40.4%)
Missing	-	20143 (15.4%)
Free School Meals Eligibility*		
Yes	13658 (25.7%)	15144 (11.6%)
No	39571 (74.3%)	38097 (29.2%)
Missing	-	77311 (59.2%)
Sex		
Male	26583 (49.9%)	67088 (51.4%)
Female	26646 (50.1%)	63041 (48.3%)
Other	-	6 (<0.1%)
Missing	-	417 (0.3%)
EAL		

Yes	18914 (35.5%)	44740 (34.3%)
No	34315 (64.5%)	81571 (62.5%)
Missing	-	4241 (3.2%)

3. Good level of development description

The primary predictor variable was the “good level of development”, a binary variable derived from the Early Years Foundation Stage Profile assessment. In this assessment, children’s skills are assessed using 17 early learning goals which cover seven areas of development (“physical development”, “communication and language”, “personal, social, and emotional development”, “mathematics”, “literacy”, “understanding the world” and “expressive arts and design”). For each early learning goal, a child’s skills can be rated as “emerging”, “expected”, “exceeding”. If the child was performing at “expected” or was “exceeding” expected levels of attainment on all early learning goals from five domains (“physical development”, “communication and language”, “personal, social, and emotional development”, “mathematics”, and “literacy”), they were said to have reached good level of development (Standards and Testing Agency, 2017). Children rated by teachers as “emerging” on any early learning goal for any of these five domains were recorded as not reaching a good Level of development.

4. Breakdown of the Ethnic Minority Sample

Ethnicity	N (%)
Asian or Asian British: Bangladeshi	1133 (4.4)
Asian or Asian British: Chinese	170 (0.7)
Asian or Asian British: Indian	1092 (4.2)
Asian or Asian British: Pakistani	15915 (61.8)
Asian or Asian British: Any other Asian background	991 (3.8)

Black: African	663 (2.6)
Black: Caribbean	91 (0.4)
Black: Any other Black background	196 (0.8)
Mixed ethnicity: White and Asian	721 (2.8)
Mixed ethnicity: White and Black African	222 (0.9)
Mixed ethnicity: White and Black Caribbean	440 (1.7)
Mixed ethnicity: Any other mixed ethnic background	507 (2.0)
Other: Arab	62 (0.2)
Other: Any other ethnic group	993 (3.9)
White: Gypsy or Irish Traveller	30 (0.1)
White: Irish	53 (0.2)
White: Any other White background	2492 (9.7)

5. R code for the logistic regression models

Equation 1: *Unadjusted logistic regression model*

glm(SEN ~ GLD, data = RQ3_post2013, family = binomial)

Equation 2: *Adjusted logistic regression model*

glm(SEN ~ GLD + EverFSMAll + ethnicity + sex + EAL, data = RQ3_post2013, family = binomial)

Equation 3: *Adjusted logistic regression model with interaction term*

glm(formula = SEN ~ GLD * ethnicity + EverFSMAll + sex + EAL, family = binomial, data = RQ3_post2013)

6. Logistic regression models to show the association between reaching a good level of development and later identification of having Special Educational Needs and how this interacts with ethnicity

Logistic regression models (SE = Standard Error; OR = Odds Ratio; CI = Confidence Intervals)

Predictor	Beta	SE	OR (95% CI)
Model 1			
GLD (Not Reached)	2.23	.02	9.34 (8.92-9.78)
Model 2			
Intercept	-2.73	.04	0.07 (0.06-0.07)
GLD (Not reached)	2.14	.02	8.50 (8.10-8.91)
FSM status (True)	0.55	.02	1.73 (1.64-1.81)
Ethnicity (White British)	0.16	.03	1.18 (1.10-1.25)
Sex (Male)	0.52	.02	1.68 (1.60-1.76)
EAL (True)	-0.03	.03	0.97 (0.91-1.03)
Model 3			
Intercept	-2.64	.04	0.07 (0.07-0.08)
GLD (Not reached)	2.05	.04	7.75 (7.23-8.30)
FSM status (True)	0.53	.02	1.70 (1.62-1.78)
Ethnicity (White British)	0.04	.04	1.04 (0.95-1.13)
Sex (Male)	0.51	.02	1.67 (1.59-1.75)
EAL (True)	-0.08	.03	0.93 (0.87-0.99)
GLD*Ethnicity	0.16	.05	1.18 (1.07-1.29)
